# Ileal Lactase Expression Associates with Lactase Persistence Genotypes

**DOI:** 10.3390/nu13041340

**Published:** 2021-04-17

**Authors:** Jan Krzysztof Nowak, Emilia Dybska, Marzena Dworacka, Natallia Tsikhan, Victoria Kononets, Saule Bermagambetova, Jarosław Walkowiak

**Affiliations:** 1 Department of Pediatric Gastroenterology and Metabolic Diseases, Poznan University of Medical Sciences, Szpitalna 27/33, 60-572 Poznan, Poland; emilia.dybska@student.ump.edu.pl (E.D.); jarwalk@ump.edu.pl (J.W.); 2 Department of Pharmacology, Poznan University of Medical Sciences, 60-572 Poznan, Poland; mdworac@ump.edu.pl; 3 Department of Pediatrics, Grodno State Medical University, 230009 Grodno, Belarus; tsikhannat@gmail.com; 4 West Kazakhstan Marat Ospanov Medical University, Aktobe 030012, Kazakhstan; micropaleontolog@yandex.kz (V.K.); b.saule74@mail.ru (S.B.)

**Keywords:** lactase persistence, lactose intolerance, lactose, milk, macrophage, monocyte

## Abstract

(1) Background: Lactose digestion depends on persistence genotypes (including rs4988235), the frequency of which exhibits broad geographical variability. However, little is known about the relationship between lactase (*LCT*) genotypes and intestinal expression of *LCT*. We aimed to investigate ileal expression of *LCT* depending on main genetic polymorphisms (rs4988235, rs3754689, rs3739022), age, sex, smoking status, body mass index (BMI), and the expression of other genes; (2) Methods: phenotype, array-based genotype, and ileal mucosal biopsy expression data were obtained from the CEDAR study; (3) Results: analyses included 196 healthy Europeans (53.6% women) aged 53.0 ± 13.6 years with a mean BMI of 25.6 ± 4.2 kg/m^2^, of whom 17.4% were smoking. Ileal *LCT* expression was mostly independent of age, sex, BMI, or smoking. Rs4988235 homozygous minor allele (GG) associated with lower *LCT* expression (vs. AG *p* = 2.2 × 10^−6^, vs. AA *p* = 1.1 × 10^−7^). Homozygous major allele of rs3754689 (GG) was related to higher *LCT* expression (vs. AG *p* = 1.7 × 10^−5^, vs. AA *p* = 0.0074). Rs3754689 genotype did not modify *LCT* expression (GG vs. AG *p* = 0.051) in rs4988235-heterozygous subgroup. Interestingly, *CD14*, which is a marker of monocytes and macrophages, was the strongest negative transcriptomic correlate of *LCT* expression (r = −0.57, p_FDR_ = 1.1 × 10^−14^); (4) Conclusions: both rs4988235 and rs3754689 associated with ileal *LCT* expression, which did not seem related to age, sex, smoking, or BMI. The inverse correlation between *LCT* and *CD14* expression in the ileum is striking and requires further investigation.

## 1. Introduction

Individuals with lactase persistence (LP) phenotype digest lactose till senility [1]. This is enabled by the sustained activity of lactase (LCT), a β-d-galactosidase also known as lactase phlorizin hydrolase (LPH) [2,3]. Its expression is observed on the brush border of the small intestine, with the highest values in jejunal microvilli [2]. Patients with non-persistence phenotype suffer from indigestion after the intake of lactose-containing products [3,4]. In this group, LCT activity usually drops after the age of five, following reduced LCT expression due to the presence of the homozygous minor allele C (G) at *LCT* position-13910 (rs4988235) [5,6].

The autosomal dominant inheritance pattern has enabled dairy intake in various populations around the world, probably reflecting adaptation to evolutionary pressures of changing lifestyles, including animal domestication [7]. The highest frequency of LP characterizes descendants of northern Europeans and some ethnic groups in Africa, which harbor distinct persistence polymorphisms [7,8,9,10,11]. LP is rarely found in South-East Asia and generally, the frequency of variants enabling lactose digestion in the adulthood falls from northwest to southeastern regions of Europe, Asia, and Africa [11].

Previous studies showed the applicability of rs4988235 genetic testing to exclude the risk of lactose intolerance in adults [8,12]. Currently, apart from standard clinical tests, the combined genetic and epigenetic assessment is advocated. DNA methylation in the *LCT* promoter region was found to associate with rs4988235 genotype, *LCT* gene expression, LCT activity, and LP [13]. Interestingly, the relationship between enzymatic activity and *LCT* mRNA level has not been confirmed in adults, even though the underlying polymorphism is known to act via a transcription regulation element [14].

*LCT* gene expression is insufficiently studied, with most investigations focusing on LP genetics and clinical assessment. This is also due to the difficulty in obtaining intestinal biopsies. In this study, we used a large ileal transcriptomic dataset from Correlated Expression & Disease Association Research (CEDAR) cohort to verify hypotheses that ileal *LCT* expression: (a) associates with *LCT* genotypes; (b) correlates with age and body mass index (BMI); (c) depends on sex and smoking status; and (d) correlates with expression of other transcripts.

## 2. Materials and Methods

**Figure 1:**
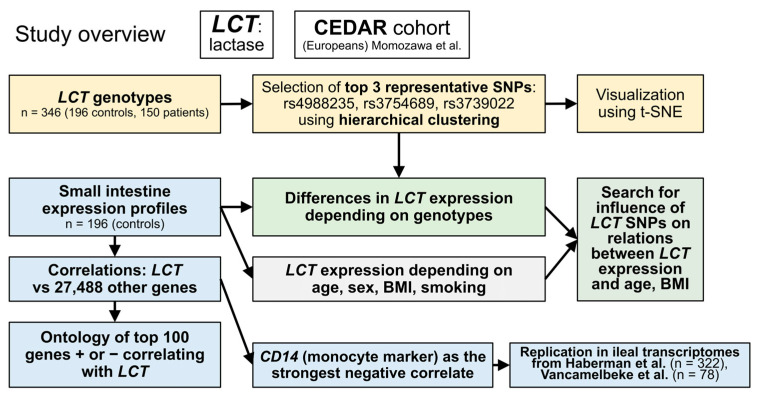
Scheme of the study. BMI: body mass index, CD14: cluster of differentiation 14, CEDAR: Correlated Expression & Disease Association Research (by Momozawa et al. [15]), LCT: lactase, SNP: single nucleotide polymorphism, t-SNE: t-distributed stochastic neighbor embedding.

CEDAR was a large study aimed at identifying expression-quantitative trait loci related to inflammatory bowel disease risk in a number of tissues, including leukocyte subsets and intestinal biopsies [15], both in healthy controls and patients with various diseases. This study focused on the healthy controls (n = 196), most of whom were middle-aged non-smokers with good nutritional status or overweight (more details in Section 3.2). The scheme of the study is presented in Figure 1.

### 2.1. Genotypes

Complete raw data (Illumina HumanOmniExpress-12 v1.0) were obtained from the Array Express (accession ID: E-MTAB-6666) and processed with GenomeStudio 2.0.5 using the adequate array design file. After clustering and quality check, SNPs from a region on chromosome 2 including the *LCT* gene with 100 kbp margin (chr2:136,445,410–136,694,750; GRCh37/hg19) were exported to a genotype matrix, which was further processed to represent the count of the major allele in the genotype. Full *LCT* locus genotype data were used to explore the genetic structure of the population by performing t-distributed stochastic neighbor embedding of study participants (t-SNE, Rtsne). Only variants with minor allele frequency (MAF) > 10% and variance > 10% were included. The obtained matrix was used for hierarchical clustering with the fastcluster package in R (R Software Foundation, Vienna, Austria), using a Ward D dissimilarity matrix. After dendrogram inspection (ggdendro) and setting a cut-off level, a most representative SNP was selected for each of the *three* resulting clusters. This choice considered minimal distance to other cluster members (representativeness) or prominence in the literature (rs4988235). The genotypes were subject to Pearson’s correlation analysis to illustrate their relationships.

### 2.2. Transcriptomic Data

Raw data from the analysis of terminal ileum mucosal RNA using Illumina’s HT12v4 expression microarray were downloaded from the Array Express (accession ID: E-MTAB-6667) and read using limma. Arrays with no variance and mean expression level of 0 were excluded, and the others underwent a quality check. The data were quantile-normalized using the qnc function, which adds an offset of 16 prior to log_2_ transformation. Since the mean log_2_ (signal + 16) from control probes was ~4.5, only genes with mean expression > 5 were included, bringing the number of available probes down from 47,323 to 27,490. Samples reporting gene expression in the terminal ileum of healthy participants were selected. The dataset obtained in this way was further used for downstream analyses. 

Two probes binding *LCT* transcripts correlated with each other (r = 0.87) and matched the same transcript with no other predicted complementary sequences in the human transcriptome (Basic Local Alignment Search Tool, BLAST) and were therefore averaged. Correlations of *LCT* with other transcripts were investigated and top genes positively and negatively associated with *LCT* were analyzed using Gene Set Enrichment Analysis biological process ontology (the Broad Institute, Cambridge, MA, USA). Phenotype data were drawn from the ArrayExpress (accession ID: E-MTAB-6667). All the statistical analyses were conducted using R directly (tidyverse*,* ggpubr) or via JASP as an interface (University of Amsterdam, Amsterdam, the Netherlands). 

A complementary correlation analysis of *LCT* and *CD14* (cluster of differentiation 14) was conducted in data from Haberman et al. [16] (Gene Expression Omnibus accession ID: GSE57945) and Vancamelbeke et al. (GSE102133) [17] directly at the R2 genomics analysis and visualization platform of the Amsterdam Medical Center (http://r2.amc.nl (accessed on 10 March 2021)). 

## 3. Results

### 3.1. Genotypes

**Figure 2:**
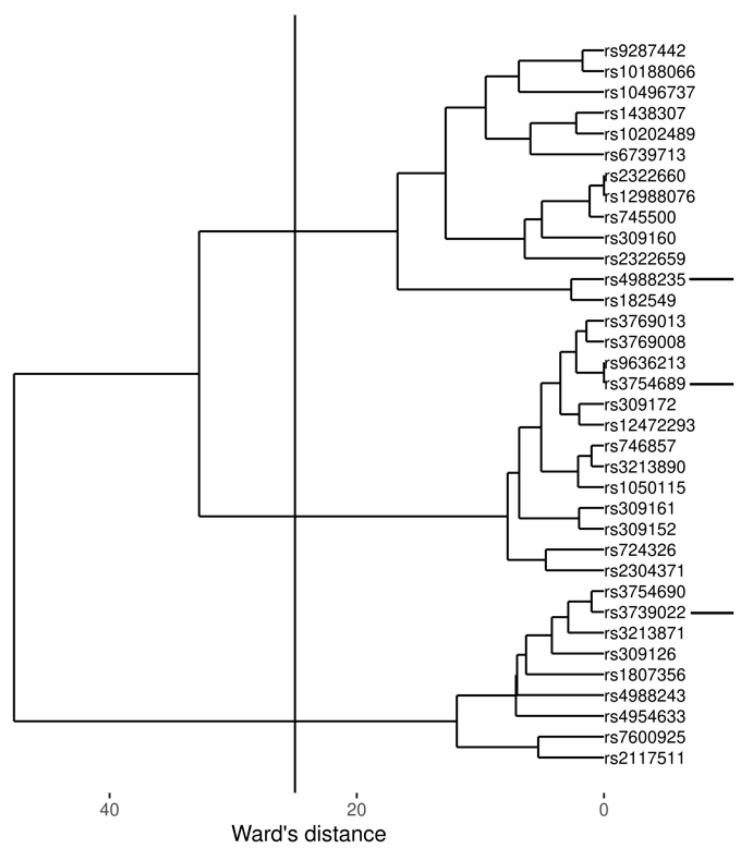
Clustering of *LCT* locus polymorphisms with minor allele frequency > 10% and variance > 10% using a Ward’s dissimilarity matrix. Three polymorphisms selected as representative for respective clusters are indicated with horizontal lines on the right.

Fifty-two SNPs were identified in *LCT* locus (±100 kbp) of 346 CEDAR participants (controls and patients), who are characterized in the original study [15]. In brief, the subjects were Europeans subject to screening colonoscopy, most of whom had no diseases but with a fraction having different diagnoses, including asthma. Seventeen polymorphisms with low MAF and variance were excluded (n = 17) and the remaining 35 were clustered using the Ward’s distance (Figure 2). 

Three clusters were identified. For each of the three clusters, a representative SNP was chosen: rs4988235, rs3754689, and rs3739022. Rs4988235 was preferred because it is the best-known variant associated with lactose intolerance. The remaining two SNPs were selected because of short distance to all other variants included in their clusters, i.e., being most representative for the largest number of polymorphisms within the clusters. It should be noted that genes in clusters were similar (e.g., correlation between rs4988235 and rs182549 was r = 0.98). Correlations between the three SNPs selected for further analysis are listed in Table 1. 

**Figure 3:**
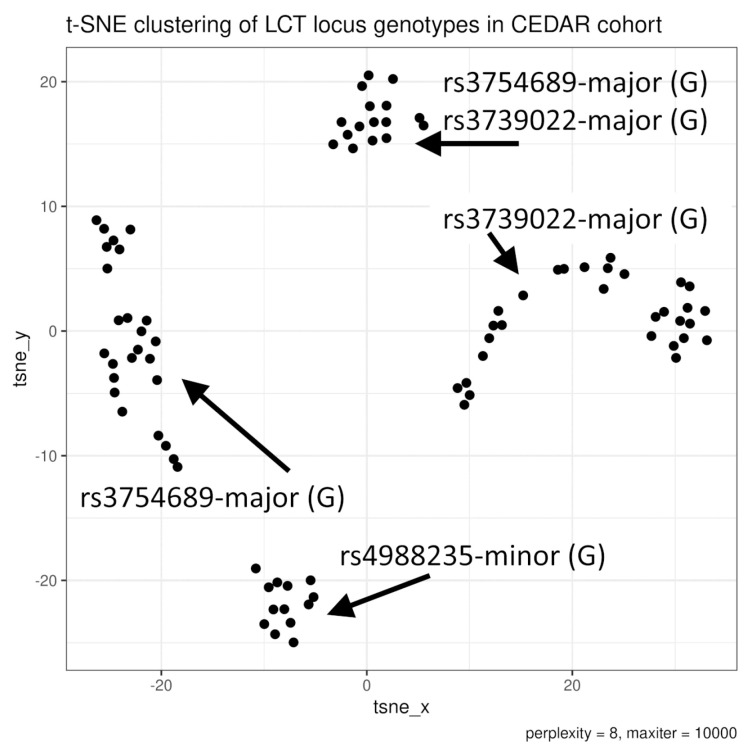
Visualization of t-distributed stochastic neighbor embedding (t-SNE) clustering of sets of *LCT* locus genotypes in CEDAR cohort based on 52 SNPs from HumanOmniExpress−12 v1.0. High prevalence of major/minor alleles is indicated.

Eighty-four CEDAR participants exhibiting unique combinations of *LCT* loci genotypes were clustered using t-SNE (Figure 3). The identified clusters exhibited specific combinations (haplotypes) of rs4988235, rs3754689, and rs3739022 genotypes, confirming that the selection of the three SNPs may be used to describe a considerable share of *LCT* locus genetic diversity. Table 2 presents allele frequencies for patients included in expression analyses. Please note that the genotypes are presented as per Illumina upper strand output, and therefore changes often known as C/T are presented herein as G/A, a convention also adopted in reports from other teams [18].

### 3.2. Expression

Terminal ileum biopsy expression data were available for 196 healthy participants of the CEDAR study. The average age ± SD was 53.0 ± 13.6 years (range 17–80 years). The group included 105 women (53.6%) and 91 men (46.4%). Most of the subjects were non-smokers (n = 162/196, 82.6%). Participants mostly exhibited good nutritional status or were overweight, with a mean body mass index (BMI) of 25.6 ± 4.2 kg/m^2^ (range 15.9–40.6).

Two probes matching *LCT* had mean expression > 5: ILMN_1749026 and ILMN_2046073. BLAST revealed that they both should bind only one and the same transcript NM_002299.2. For this reason and because of a high degree of correlation (r = 0.87), the levels of the two probes were averaged.

Ileal *LCT* did not significantly correlate with age (r = −0.10, *p* = 0.169) or BMI (r = −0.055, *p* = 0.445). It also did not depend on sex (female 6.22 ± 1.32 vs. male 6.09 ± 1.16, Mann-Whitney *p* = 0.677) or smoking status (smoker 6.00 ± 1.10 vs. non-smoker 6.19 ± 1.28, *p* = 0.505).

The top transcriptomic correlates of the expression of *LCT* are reported in Table 3. Interestingly, the expression of *CD14*, which is a marker of monocytes and macrophages, was the strongest negative correlate of *LCT* (r = −0.57, p_FDR_ = 1.1 × 10^−14^). *LCT* expression most strongly positively associated with mitochondrial acylating methylmalonate-semialdehyde dehydrogenase (r = 0.56, p_FDR_ = 2.4 × 10^−14^). Other noteworthy correlates of *LCT* included *TLR3* (toll-like receptor 3; r = 0.51, *p* = 2.4 × 10^−11^), *RORC* (RAR-related orphan receptor C; r = 0.49, *p* = 7.4 × 10^−11^), and *ACE2* (angiotensin converting enzyme 2; r = 0.47, *p* = 3.8 × 10^−10^), all of which attract attention in the context of inflammatory bowel disease. ACE2 is also known to enable cellular entry of the severe acute respiratory syndrome coronavirus (SARS-CoV-2) [19]. Full results of correlation analysis are presented in Table S1 (supplement). 

The negative correlation between *LCT* and *CD14* was confirmed in a dataset of 322 ileal biopsies from patients with inflammatory bowel diseases and healthy controls (r = −0.43, *p* = 6.78 × 10^−16^) by Haberman et al. [16] and in 78 ileal biopsies from patients with Crohn’s disease and controls obtained by Vancamelbeke et al. (r = −0.55, *p* = 1.83 × 10^−7^) [17].

**Figure 4:**
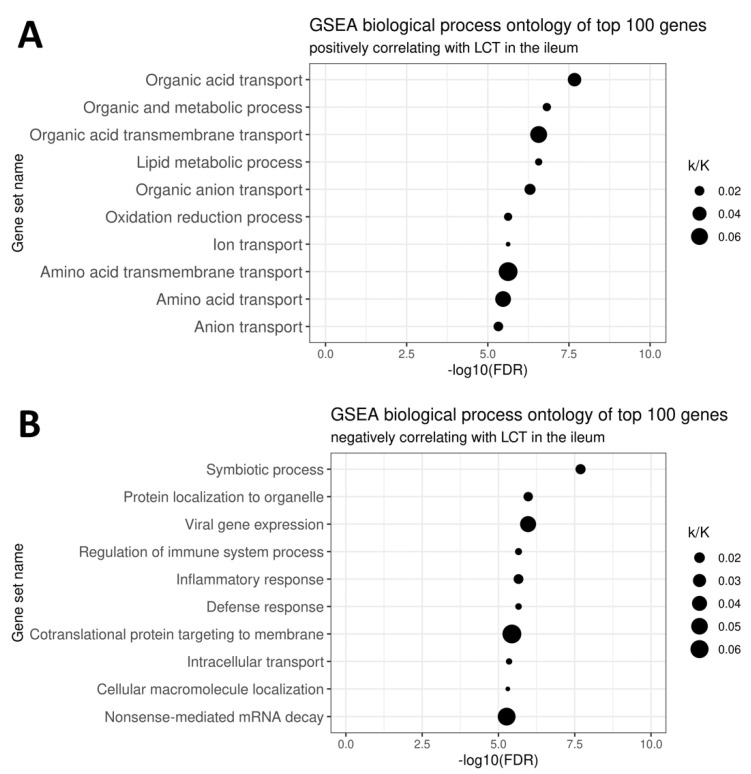
Gene set enrichment analysis of top 100 genes positively (**A**) and negatively (**B**) correlating with *LCT*. The k/K ratio indicates what fraction of all the genes in a given set overlapped with the investigated list of 100 genes (e.g., out of 353 genes in the Organic acid transport gene set, 13 were also found among genes most strongly correlating with *LCT*, yielding k/K = 0.037).

Principal results of gene ontology analysis of transcripts positively and negatively correlating with *LCT* is shown in Figure 4. Genes positively correlating with *LCT* were involved in nutrient transmembrane transport and metabolism. On the other hand, genes negatively associated with *LCT* related to symbiosis, inflammatory reactions, and protein trafficking.

### 3.3. Relations between LCT Genotype and Expression

**Figure 5:**
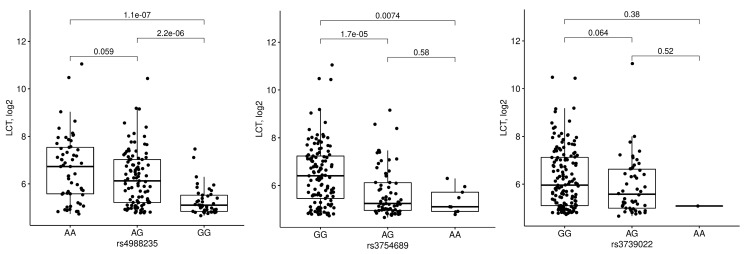
Ileal *LCT* expression depending on *LCT* locus single nucleotide polymorphisms.

**Figure 6:**
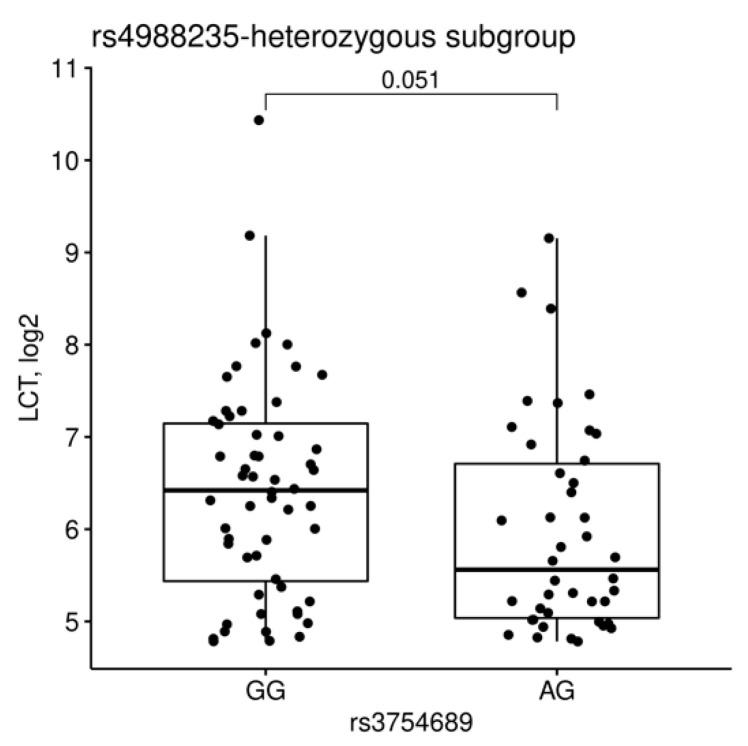
Ileal *LCT* expression depending on rs3754689 genotype in a subgroup of rs4988235-heterozygous (AG) CEDAR study participants.

Expression of *LCT* was dependent on rs4988235 and rs3754689 genotypes (Figure 5). Comparisons of homozygous major (G) vs. heterozygous rs3739022 alleles revealed no statistically significant differences (*p* = 0.064). Lower *LCT* expression was therefore associated only with G allele of rs4988235 and A allele of rs3754689. To verify if rs3754689 may modify the relationship between rs4988235 and *LCT*, analysis was performed in a subgroup heterozygous for rs4988235. The analysis did not reveal statistically significant differences, although a larger sample size would probably be required to determine whether such relationship exists (Figure 6). 

**Figure 7:**
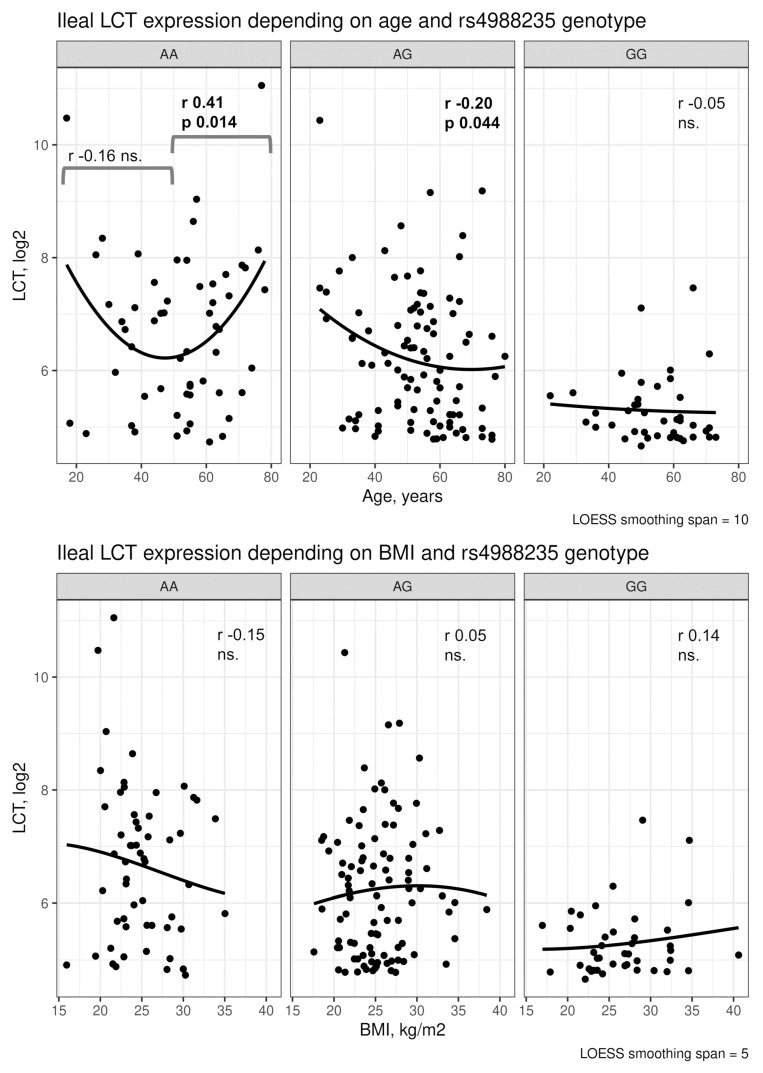
Relationships of ileal *LCT* expression vs. age and BMI depending on the lactase persistence polymorphism rs4988235. Pearson’s r is shown in the case of AA genotype correlation analysis was conducted separately in patients younger and older than 50 years old due to the appearance of a U-shaped pattern.

Further analyses revealed that rs4988235 might modify the relationship between *LCT* expression and age in a non-linear manner (Figure 7). Whereas in patients with GG and AG genotypes the *LCT* vs. age relationship seemed linear, in the AA group it appeared U-shaped, with greater *LCT* expression in early adulthood and in the old age. This effect, however, appeared to be driven by outliers and would require additional verification. No similar effects were be found for BMI.

## 4. Discussion

In this work, we described genomic and transcriptomic characteristics of lactase persistence status in the ileal mucosa of 196 controls from the CEDAR cohort [15]. Two representative SNPs related to ileal *LCT* gene expression: rs4988235 (major allele A) and rs3754689 (major allele G). No strong and consistent relationships were found for age, sex, BMI, or smoking status. A thought-stimulating negative correlation between *LCT* and *CD14* was found.

### 4.1. The Role of rs4988235 and rs3754689

The robust association between rs4988235 (C/T -13910) and lactase persistence in Europeans is well-known [8,12,20]. The A allele maintains high *LCT* activity, and thus enables cow’s milk digestion in adulthood [20]. The A allele carriers have a greater mucosal expression of *LCT* in the intestine [21]. Our observations confirm this genetic regulation of *LCT* transcription. 

In contrast, rs3754689 has been poorly described in the context of LP. It was investigated as one of the *LCT* haplotype markers, but was not strongly associated with lactose intolerance [22]. It related, however, to a lower risk of inhibitor development in patients with hemophilia A [23]. The variant rs3754689 requires further investigation to better understand its potential clinical value.

### 4.2. Ileal LCT Expression and Clinical Characteristics

Depending on the ethnicity, up to 70% of adults suffer from lactose intolerance as a result of a progressive decrease in intestinal lactase activity [11]. Genotyping rs4988235 (and the closely correlated rs182549) is considered to enable diagnosis of lactose intolerance in symptomatic patients older than six years [24] and may be considered to have accuracy increasing with age [3]. Nevertheless, the relationship with clinical status [6] or LP phenotype [13] is not strict. Epigenetic changes accumulate with age and may contribute to markedly different DNA regulatory landscapes [25,26]. These probably provide a variety in the programmed ability to digest lactose-rich dairy products. Our study did not investigate gene methylation, nor did it confirm the general (across genotypes) significance of age on *LCT* expression in the investigated group of European adults. However, high *LCT* expression constituted a common feature of rs4988235 AA-homozygotes in early adulthood and the old age. Further analyses are needed to check whether the U-shaped effect (which seemed mostly outlier-driven) can be confirmed in Europeans or found in other ethnic groups.

The ability to digest milk affects dietary habits in adulthood. Consumption of lactose-containing dairy products without risk of diarrhea or other symptoms may associate with a more varied diet, and thus distinct body composition. Genome-wide association studies indicated rs4988235 as associated with obesity [27,28,29], which can be explained by a lifetime exposure pattern to cow’s milk and fat-rich dairy products [27,28,29]. However, this study did not confirm the relationship between ileal *LCT* expression and BMI. Neither smoking status nor sex-specific analyses provided any associations with the *LCT* variants, reinforcing the observation by Kettunen et al. of no gender impact on BMI in LP individuals [27].

### 4.3. Transcriptomic Correlates of Ileal LCT Expression Including CD14

Besides the ileal *LCT* genotype-expression relationships, we also explored its transcriptomic correlates. The top correlate (and a negative one) of *LCT* was the key monocyte marker *CD14*. Previously, expression of *LCT* (dependent on rs370868556), was related to the white blood cell pool [30]. It might be speculated that high LCT expression reduces distension of the bowel wall, contributing to less tissue stress that could attract CD14+ cells. On the other hand, attracted CD14+ cells could potentially damage villi and contribute to lactase deficiency. An association with *RNASET2* (ribonuclease T2) might indicate a reduced need to recognize pathogen RNAs, which could be related to antiviral activity of whey protein [31]. The lactose/monocyte axis was previously associated with the upregulation of gastrointestinal antimicrobial peptides such as cathelicidin antimicrobial peptide (CAMP) and thus modulated mucosal immunity in the intestine [32]. Such relationships could intervene in the pathogenesis of small intestinal bacterial overgrowth. The correlation analyses described above indicate interesting new research themes at the interface between LP and immunity.

Other top negative correlates of *LCT* expression also included cyclin CDC37 (cell division cycle 37), which stabilizes LKB1 (liver kinase B1), the protein deficient in Peutz-Jeghers syndrome. There are, however, no data available on putative relationships between lactose digestion or milk consumption and outcomes in this genetic disease. The inverse association between hexokinase 1 and *LCT* expression may indicate shifts in mucosal carbohydrate processing.

*LCT* expression associated with metabolism-related *ALDH6A1* (aldehyde dehydrogenase 6 family member A1), which contributes to downregulation of the acetyl-CoA pathway. Such processes lead to adipose tissue changes in diabetic obese individuals [33]. A positive correlation between *LCT* and *ALDH6A1* may suggest a link between LP and adiposity, possibly mediated by microbiota [18], which could be further investigated. Another positive correlate of *LCT* expression was *XYLB*, encoding xylulokinase involved in energy metabolism, which we speculate might be related to small intestinal bacterial overgrowth. Interestingly, *CISD1* (CDGSH iron sulfur domain 1) also associated with *LCT;* CISD1 is a mitochondrial protein reduced in cystic fibrosis, the deficiency of which may cause Wolfram syndrome with neurodegeneration [34]. Apparently, higher *LCT* expression correlates with enhanced energy metabolism and nutrient transport.

### 4.4. Generalization and Limitations

Data obtained from the CEDAR study provide insight into the genetics of *LCT* locus in Europeans. They present two key advantages: a large cohort and gene expression profiling in intestinal biopsies. This foundation allowed us to conduct an ileum-focused analysis, determine *LCT* ileal expression, and identify novel *LCT* correlates. Dietary habits, especially daily milk intake, are lacking factors in our analyses. Moreover, no information on microbiota was available. It is necessary to reinstate that *LCT* gene expression is not synonymous with lactase enzyme activity. Data obtained from Europeans should not be generalized to the global population as not only the frequency of lactase persistence but also its epigenetic regulation may undergo population-specific fine-tuning. The results should also not be generalized to children. Finally, results from omics analyses may considerably change depending on the overall approach and methods. 

## 5. Conclusions

To conclude, our study indicates a positive correlation between major A allele of rs4988235 as well as major G allele of rs3754689 and *LCT* gene expression in ileal mucosa. Ileal *LCT* expression does not seem related to age, sex, smoking, or BMI. Potential modification of the relationship between rs4988235 and LP by rs3754689 appears as an interesting research topic. The inverse correlation between *LCT* and *CD14* expression in the ileum is striking and requires further investigation, especially in the context of irritable bowel syndrome, small intestinal bacterial overgrowth, and cow’s milk protein allergy.

## Figures and Tables

**Table 1:** Correlations between analyzed polymorphisms representative of genetic variance in *LCT* locus.

SNP Pair	Pearson’s r	*P*
rs4988235–rs3754689	0.60	2.2 × 10^−16^
rs4988235–rs3739022	0.38	2.6 × 10^−13^
rs3754689–rs3739022	−0.18	6.8 × 10^−4^

**Table 2:** *LCT* locus allele frequency in healthy CEDAR participants with paired ileal expression data.

Polymorphism	Homozygous Major	Heterozygous	Homozygous Minor
rs4988235	AA 57 (29.1%)	AG 98 (50.0%)	GG 41 (20.9%)
rs3754689	GG 121 (61.7%)	AG 66 (33.7%)	AA 9 (4.6%)
rs3739022	GG 141 (72.3%)	AG 50 (25.6%)	AA 1 (0.5%)

**Table 3:** Pearson’s correlations between mean *LCT* expression and other transcripts in the terminal ileum of healthy participants of CEDAR study.

Gene	r	p_FDR_
Positively correlating with *LCT* expression		
*ALDH6A1—*Aldehyde Dehydrogenase 6 Family Member A1	0.56	2.4 × 10^−14^
*LOC441442*	0.56	8.9 × 10^−14^
*C18orf18—*Long Intergenic Non-Protein Coding RNA 526	0.55	2.5 × 10^−13^
*XYLB—*Xylulokinase	0.55	2.8 × 10^−13^
*CISD1—*CDGSH Iron Sulfur Domain 1	0.54	6.3 × 10^−13^
*BCL2L15—*BCL2 Like 15	0.54	6.5 × 10^−13^
*LOC100128907*	0.54	8.3 × 10^−13^
*CCDC25—*Coiled-Coil Domain Containing 25	0.54	8.9 × 10^−13^
*BEND7—*BEN Domain Containing 7	0.53	2.3 × 10^−12^
*APOM—*Apolipoprotein M	0.53	3.2 × 10^−12^
Negatively correlating with *LCT* expression		
*CD14—*Monocyte Differentiation Antigen CD14	−0.57	1.1 × 10^−14^
*RNASET2—*Ribonuclease T2	−0.50	2.9 × 10^−11^
*CDC37—*Cell Division Cycle 37, HSP90 Cochaperone	−0.50	3.6 × 10^−11^
*C7orf50—*Chromosome 7 Open Reading Frame 50	−0.50	5.4 × 10^−11^
*LOC730187*	−0.49	6.3 × 10^−11^
*UBE2I—*Ubiquitin Conjugating Enzyme E2 I	−0.49	1.0 × 10^−10^
*LOC650369*	−0.49	1.0 × 10^−10^
*RCC2—*Regulator of Chromosome Condensation 2	−0.49	1.0 × 10^−10^
*HK1—*Hexokinase 1	−0.49	1.1 × 10^−10^
*RPL27—*Ribosomal Protein L27	−0.48	2.3 × 10^−10^

## Data Availability

Data are publicly available in the Array Express (E-MTAB-6666 and E-MTAB-6667) and Gene Expression Omnibus (GSE57945 and GSE102133).

## Supplementary Materials

The following are available online at https://www.mdpi.com/article/10.3390/nu13041340/s1, Table S1: Full results of *LCT* correlation analysis in CEDAR ileal biopsies from healthy controls.
